# The effect of Bushenhuoxue nutritional decoction in the treatment of overweight patients with polycystic ovarian syndrome

**DOI:** 10.12669/pjms.40.6.9304

**Published:** 2024-07

**Authors:** Jiaowen Fu, Yingqi Chen, Zongwen Huang, Xiaojing Wang

**Affiliations:** 1 Jiaowen Fu, Department of Traditional Chinese Medicine, The First Affiliated Hospital of Hainan Medical University, Haikou, Hainan Province 570102, P.R. China; 2 Yingqi Chen, Department of Chinese medicine prescription, Hainan Medical University, Haikou, Hainan Province 571199, P.R. China; 3 Zongwen Huang, Department of Traditional Chinese Medicine, The First Affiliated Hospital of Hainan Medical University, Haikou, Hainan Province 570102, P.R. China; 4 Xiaojing Wang, Department of Clinical Nutrition, The First Affiliated Hospital of Hainan Medical University, Haikou, Hainan Province 570000, P.R. China

**Keywords:** Traditional Chinese medicine, Polycystic ovarian syndrome, Nutrition support, Metabolism, Hormone level

## Abstract

**Objective:**

To assess the effect of Traditional Chinese Medicine (TCM) nutrition treatment (Bushenhuoxue nutritional decoction) in overweight patients with polycystic ovary syndrome (PCOS).

**Methods:**

Retrospective analysis of 96 overweight patients with PCOS who received treatment in our hospital from October 2020 to June 2022 was done. Among them, 46 patients received routine drug treatment and daily dietary intervention (control group), while 50 patients received additional TCM nutrition support in addition to routine treatment (observation group). Glucose and lipid metabolism indicators and hormone levels were compared between the two groups before and after the treatment. Ovulation rate, pregnancy rate, and adverse reactions were compared between both groups one year after the treatment.

**Results:**

After treatment, the improvement of glucose and lipid metabolism indicators and hormone levels in the observation group was significantly better than in the control group (*P*<0.05). After treatment, the TCM syndrome scores of the two groups were lower than that before treatment (P < 0.001), and the TCM syndrome scores of the observation group was lower than that of the control group (P < 0.001).Ovulation and pregnancy rates were significantly higher in the observation group compared to the control group at 1-year follow up (*P*<0.05), and the incidence of adverse reactions in the observation group was significantly lower than that in the control group (*P*<0.05).

**Conclusions:**

Combined with conventional drug treatment, TCM nutrition treatment can significantly improve glucose and lipid metabolism, hormone levels, and TCM syndrome of overweight PCOS patients, increase the ovulation and pregnancy rates, and reduce potential adverse reactions.

## INTRODUCTION

Polycystic ovary syndrome (PCOS) is a common endocrine and metabolic disorder in women of childbearing age with an incidence of 8% -13%.^1^,^2^ The main manifestations of PCOS include menstrual irregularities, hirsutism, acne, infertility, obesity, glucose metabolism disorders, and ovarian dysfunction that impact ovulation and seriously affect reproductive function and overall health of the patients.^2^ According to the Traditional Chinese Medicine (TCM), PCOS is the main factor leading to infertility in young women,^3^ and the main cause of infertility caused by PCOS is located in the uterus and involves kidneys, spleen, and liver.^4^ The main pathogenesis of ovulatory dysfunction infertility caused by PCOS, according to TCM, is spleen and kidney yang deficiency, combined with various syndromes such as phlegm dampness, liver depression, and blood stasis.^5^,^6^ In terms of treatment, the main approach should be, therefore, to warm the spleen and tonify the kidney, while also using methods such as dispelling phlegm, soothing the liver, and resolving blood stasis.^7^-^9^

TCM nutrition is a fusion of physiology and pathology under the guidance of basic theories of TCM. This innovative dietary therapy and auxiliary medical method organically combines dialectical diet with modern nutrition.^8^,^9^ There are currently no literature reports on the treatment of overweight PCOS patients using TCM nutrition. Therefore, this study retrospectively analyzes the clinical efficacy of TCM nutrition treatment in overweight patients with PCOS to provide reference for future clinical interventions.

## METHODS

Medical records of 96 patients with PCOS who received treatment in our hospital from October 2020 to June 2022 were retrospectively analyzed. A total of 46 patients in the cohort received conventional drug treatment and nutritional intervention management were set as the control group, while 50 cases patients received TCM nutrition treatment in addition to conventional drug treatment were assigned to the observation group.

### Inclusion criteria:

- Patients met the western diagnosis of PCOS.^1^
- Patients with the following primary symptoms and ≥2 secondary symptoms with tongue and pulse symptoms met the TCM diagnosis of PCOS: (1) *Primary symptoms:* irregular menstruation or amenorrhea; (2) *Secondary symptoms:* abdominal distension, diarrhea, heaviness and drowsiness in head and body, or obesity; (3) *Tongue and pulse symptoms:* pale tongue, white greasy moss, moist and slippery pulse or slippery pulse.
- BMI ≥ 25kg/m^2^.
- Patients with normal sexual activity that having been infertile for at least one year.
- The clinical data is complete.

### Exclusion criteria:

- Patients with combined dysfunction of important organs.
- Patients with mental illness.
- Presence of other serious primary or secondary diseases.
- Patients with irregular menstruation caused by other endocrine diseases such as thyroid dysfunction.
- Patients with history of hormone therapy within the past three months.

### Ethical Approval

This study has been approved by the Ethics Committee of our hospital (Approval No.: 2022-L-135; date: August 3, 2022).

### Conventional drug treatment and daily dietary intervention

Routine drug treatment and nutritional intervention were given by the medical staff based on the daily diet of the patients. Patients were guided to consume high-protein, low-fat, and low-sugar foods, combined with moderate aerobic exercise for at least 40 minutes each time. From the first day of menstruation, patients were administered metformin (Shenzhen Zoomlion Pharmaceutical Co., Ltd., Approval No: H44024853; 0.25g/tablet), 0.5g/time, three times/day. At the same time, dietary intervention and psychological counseling were provided to the patients, and the patient’s diet and exercise status were recorded weekly. The course of treatment was 12 weeks.

### TCM nutrition (Bushenhuoxue nutritional decoction)

Nutritional support was provided along with metformin treatment, integrated the holistic dialectical diet of TCM and the theory of nutritional science. Specifically, it referred to the provision of TCM nutrition service programs based on dietary conditioning, Tonic Diet therapy and medication. TCM nutrition included nutritional soup (one chicken foot, 5g turtle shell, 40g yam, 20g black soybean, 20g black sesame, 5g goji berries, 3g orange peel, 5 big dates, and 10 hours of boiling), specific nutritional guidelines (staple food, vegetables, fruits, high protein and low-carbon water), heat distribution ratio (morning, lunch, and evening ratio 3:4:3), and suggestions from the perspective of lifestyle intervention (daily life, reasonable diet, exercise and massage). The course of treatment was 12 weeks.

### Outcome measures:

### Glucose metabolism indicators

Fasting plasma glucose (FPG) and two hours postprandial blood glucose (2hPBG) were measured using Hitachi 7600 fully automatic biochemical analyzer (Hitachi, Japan) and respective reagent kit; fasting insulin (FINS) was measured using electrochemical immunoluminescence (Roche, Switzerland). Homeostasis model assessment of insulin resistance (HOMA-IR) was calculated as HOMA-IR=FBG × FINS/22.5.^10^

### Lipid metabolism indicators

Total cholesterol (TC), High-density lipoprotein (HDL-C), low-density lipoprotein (LDL-C), triglyceride (TG) was measured using Hitachi 7600 fully automatic biochemical analyzer (Hitachi, Japan) and appropriate reagent kit.

### Hormone indicators

Follicle stimulating hormone (FSH), Luteinizing hormone (LH), estrogen (E_2_), testosterone (T) was measured by radioimmunoassay. The kit was purchased from Shenzhen Jingmei Biotechnology Co., Ltd. Ovulation rate and pregnancy rate were analyzed after one year.

### TCM syndrome scores

A TCM syndrome scale was developed for scoring patients’ primary, secondary, and tongue and pulse syndromes before and after treatment.^11^

### Adverse reactions

Gastrointestinal reactions, skin adverse reactions, hypoglycemia were recorded after treatment.

### Statistical Analysis

SPSS 22.0 (IBM Corp, Armonk, NY, USA) was used for statistical analysis. The measurement data that met the normal distribution were represented by(*χ̅*±*S*) The inter group comparison used independent sample *t* test, and the intra group comparison used paired *t* test. Data that did not meet the normal distribution were expressed by M (IQR), and the Mann-whitney *U* test was used for comparison between the groups. Counting data were expressed by n (%), and Chi-squared test was used for comparison between the groups. *P*<0.05 indicated that the difference is statistically significant.

## RESULTS

A total of 96 patients were included in this retrospective study, 46 patients in the control group and 50 patients in the observation group. The age of the patients ranged from 20 to 40 years, with a mean age of 29.78±5.47 years. Body mass index (BMI) range was 25.06~35.64kg/m2, with a mean of 29.52±3.00kg/m^2^. There was no significant difference in the age, BMI, waist–hip ratio and pregnancy history between the two groups (*P*>0.05), Table-I.

**Table-I:** Comparison of baseline data between the two groups.

Group	n	Age (years)	BMI (kg/m^2^)	Waist–hip ratio	Pregnancy history (%)	

					No	Yes
Control group	46	30.28±5.56	28.83 (26.88, 30.71)	0.91±0.12	37(80.4)	9(19.6)
Observation group	50	29.48±5.47	30.06 (27.21, 32.56)	0.90±0.13	43(86.0)	7(14.0)
*X^2^/t/z*		0.713	-1.415	0.441	0.534	
*P*		0.478	0.157	0.66	0.465	

Before the treatment, there was no statistically significant difference in FPG, 2hPBG, FINS, and HOMA-IR between the two groups (*P*>0.05). After the treatment, all glucose metabolism indicators significantly decreased in both groups compared to pretreatment level, Table-II.

**Table-II:** Changes in glucose metabolism indicators in the two groups.

Indicators	Time	Control group (n=46)	Observation group (n=50)	t	P
FPG(mmol/L)	Before treatment	4.87±1.47	4.94±1.41	-0.242	0.809
	After treatment	4.30±1.06^*^	3.83±1.03^*^	2.221	0.029
2hPBG(mU/L)	Before treatment	7.30±1.39	7.48±1.03	-0.715	0.476
	After treatment	5.69±1.08^*^	5.15±1.21^*^	2.309	0.023
FINS(mmol/L)	Before treatment	19.26±1.92	19.16±2.37	0.224	0.823
	After treatment	8.50±1.21^*^	7.91±1.06^*^	2.550	0.012
HOMA-IR	Before treatment	4.08±1.40	4.24±1.41	-0.569	0.571
	After treatment	1.61±0.38^*^	1.37±0.44^*^	2.810	0.006

***Note:*** Compared with the same group before treatment,

* *P*<0.05.

Before treatment, there was no statistically significant difference in lipid metabolism indicators between the two groups (*P*>0.05). After the treatment, TC levels in the control group decreased significantly compared to pretreatment levels (*P*<0.05). TCM nutrition treatment was associated with a significant decrease in TC, LDL-C, and TG, and a significant increase in HDL-C compared to the pretreatment levels (*P*<0.05). After the treatment, the differences in various indicators between the two groups were statistically significant (*P*<0.05), Table-III.

**Table-III:** Changes in blood lipid metabolism indicators in the two groups.

Indicators	Time	Control group (n=46)	Observation group (n=50)	t	P
TC (mg/dL)	Before treatment	201.91±15.92	206.40±19.63	-1.224	0.224
	After treatment	176.76±17.77^*^	153.73±22.01^*^	5.609	<0.001
HDL-C (mg/dL)	Before treatment	42.00±6.54	41.89±7.20	0.079	0.937
	After treatment	42.78±5.08	47.23±7.02^*^	-3.579	0.001
LDL-C (mg/dL)	Before treatment	124.85±14.41	126.16±12.51	-0.473	0.637
	After treatment	120.93±13.47	106.62±10.49^*^	5.833	<0.001
TG (mg/dL)	Before treatment	149.47±15.29	145.09±13.85	1.474	0.144
	After treatment	142.84±17.72	136.38±10.39^*^	2.155	0.035

***Note:*** Compared with the same group before treatment,

* P<0.05.

Before treatment, there was no statistically significant difference in hormone indicators between the two groups (*P*>0.05). After the treatment, the levels of FSH in both groups significantly increased, while the average levels of LH, E_2_, and T-water significantly decreased (*P*<0.05). The differences in various indicators between the two groups were statistically significant (*P*<0.05) Table-IV.

**Table-IV:** Changes in hormone indicators in the two groups.

Indicators	Time	Control group (n=46)	Observation group (n=50)	*t*	*P*
FSH(mU/mL)	Before treatment	3.42±0.43	3.42±0.43	-0.074	0.941
	After treatment	4.21±0.50^*^	5.56±0.43^*^	-14.046	<0.001
LH(mU/mL)	Before treatment	13.55±1.72	13.17±1.64	1.105	0.272
	After treatment	10.41±1.55^*^	7.60±1.51^*^	9.007	<0.001
E_2_(pg/mL)	Before treatment	1.83±0.29	1.89±0.26	-1.238	0.219
	After treatment	1.26±0.30^*^	1.06±0.21^*^	3.929	<0.001
T(ng/dL)	Before treatment	17.49±2.99	17.38±2.14	0.211	0.834
	After treatment	14.43±2.44^*^	12.58±2.19^*^	3.915	<0.001

***Note:*** Compared with the same group before treatment,

* P<0.05.

After one year of follow-up, the ovulation rate in the observation group was 80.0%, significantly higher than 56.6% in the control group (*P*<0.05). The pregnancy rate in the observation group was 62.0%, significantly higher than the 28.3% in the control group (*P*<0.05) Table-V.

**Table-V:** Comparison of ovulation rate and pregnancy rate between the two groups .

Group	n	Ovulation rate (%)		Pregnancy rate (%)	

		No	Yes	No	Yes
Control group	46	20(43.5)	26(56.5)	33(71.7)	13(28.3)
Observation group	50	10(20.0)	40(80.0)	19(38.0)	31(62.0)
*χ* ^2^		6.147		10.985	
*P*		0.013		0.001	

Before treatment, there was no statistically significant difference in TCM syndrome scores between the two groups (*P*>0.05). After treatment, the TCM syndrome scores of the two groups were lower than that before treatment (P < 0.001), and the TCM syndrome scores of the observation group was lower than that of the control group (P < 0.001). Table-VI. The incidence of adverse reactions in the observation group was 20.0%, significantly lower than the 39.1% in the control group (*P*<0.05) Table-VII.

**Table-VI:** Comparison of TCM syndrome scores before and after treatment between the two groups.

Time	Control group (n=46)	Observation group (n=50)	Z	P
Before treatment	34.00(26.75, 35.00)	34.50(30.00, 36.00)	-1.872	0.061
After treatment	14.00 (12.00, 16.00)	9.00 (7.00, 10.00)	-6.731	<0.001
*Z*	-6.217	-5.989		
*P*	<0.001	<0.001		

**Table-VII:** Comparison of Adverse Reaction Incidence between the Two Groups.

Group	n	Gastrointestinal reactions (%)	Skin adverse reactions (%)	Hypoglycemia (%)	Total occurrence rate (%)
Control group	46	12(26.1)	3(6.5)	3(6.5)	18(39.1)
Observation group	50	7(14.0)	2(4.0)	1(2.0)	10(20.0)
*χ* ^2^					4.244
*P*					0.039

## DISCUSSION

This study compares the clinical efficacy of TCM nutritional therapy and conventional nutritional intervention in combination with conventional drug therapy in treating overweight patients with PCOS. Our results showed that TCM nutrition treatment can significantly improve glucose and lipid metabolism and hormone levels, promote ovulation, increase the probability of pregnancy, and reduce adverse reactions to medication in overweight PCOS patients.

PCOS is characterized by the long course of the disease and recurrent attacks. PCOS patients usually have endocrine disorders syndrome characterized by insulin resistance and obesity.^12^ The main clinical treatment measures for PCOS currently include medication to promote ovulation, regulate menstruation, and restore pregnancy.^13^ However, overweight and obese PCOS patients have more severe endocrine and reproductive metabolic disorders, which can easily affect the efficacy of monotherapy.^12^,^13^ Neves et al.^14^ showed that early lifestyle intervention and nutritional treatment for PCOS patients can effectively reduce weight, improve lipid metabolism, control blood sugar, promote ovulation, and increase pregnancy rates in PCOS patients.

Metformin can reduce the production and intestinal absorption of glucose, and overcome insulin resistance by increasing the uptake and utilization of peripheral glucose and improving insulin sensitivity.^15^ Current studies also show that metformin is moderately efficient in reducing body weight of overweigh and obese patients.^16^ Body weight reduction was reported to restore physical health and reproductive function of PCOS patients.^15^,^17^ For instance, Zhang et al.^18^ showed that weight loss of 6% -7% can restore normal ovulation in some PCOS patients. These studies further strengthening the notion that timely metformin treatment may be beneficial in overweight PCOS patients. However, Yendapally et al.^19^ reported that the use of metformin had a negative impact on the cardiovascular and endocrine system, and was associated with adverse gastrointestinal reactions, liver and kidney dysfunction, etc. In addition, accumulation of metformin may lead to serious lactic acidosis. By adjusting the patient’s diet and developing personalized nutritional interventions, clinicians may, therefore, help patients to reduce weight while minimizing adverse reactions caused by the medication.^17^,^19^

The results of our study showed that glucose metabolism indicators of patients in both groups after the treatment were lower than before the treatment, and significantly lower in patients who received TCM dietary support compared to the control group. This indicates that TCM nutritional therapy can significantly improve the glucose metabolism level of overweight PCOS patients. The study by Ganie MA et al.^20^ has shown that most PCOS patients have abnormal glucose metabolism. Therefore, it is plausible that reducing total energy intake, optimizing the proportion of protein intake, and reducing the carbohydrate intake may contribute to improving patient prognosis.^20^

The results of this study also showed that TCM nutritional therapy significantly improved the levels of lipid metabolism indicators. Our results confirm previous studies that report that patients, who received TCM nutritional support, had improved glucose metabolic indexes and reported some degree of weight loss.^21^,^22^ Since there is a close relationship between sugar metabolism and lipid metabolism, our results further confirm that TCM nutritional therapy may have a significant effect on both glucose and lipid metabolism.

The results of this study showed that after the treatment, the hormone indicators in the observation group improved significantly compared to the control group. This indicates that TCM nutritional therapy can significantly improve hormone levels in overweight patients with PCOS. At the same time, ovulation and pregnancy rates in the observation group were significantly higher than those in the control group after one year. This indicates that TCM nutrition therapy can also alleviate menstrual irregularities in PCOS patients, promoting ovulation, and increasing their chances of conception.

We showed that the incidence of adverse reactions in the observation group was significantly lower than that in the control group. Our results indicate that TCM nutritional therapy may have a more reliable and safe therapeutic effect compared to conventional drug therapy. Metformin, as a basic drug for the treatment of overweight PCOS patients, often causes various adverse reactions.^19^ TCM nutritional therapy starts with the patient’s kidney, liver, and heart, achieving the principle of treating the root cause of the disease.^8^,^9^ According to the nutrition principle of TCM, food materials should be selected reasonably to assist the use of herbs for nourishing the kidney and liver, increase the body autoregulation and compensatory effect, and improve the overall physical condition of the patient.^23^ The nutrition in the TCM preparation of the current study have the effect of nourishing kidneys, liver and activating blood circulation. Turtle shell nourishes kidneys and liver^24^; Chinese yam nourishes kidneys, lung and spleen^25^; black soybean and black sesame nourishes liver, kidneys, blood, and moistens dryness^26^; goji berries and big dates nourishes and invigorates the blood; orange peel ameliorates diabetic nephropathy^27^. We speculate that this is also the reason that the TCM syndrome scores improved in this study. Compared to conventional nutritional intervention, TCM nutritional therapy has the advantage of accurately reducing the adverse effects of Western medicine, providing patients with nutrition, and thereby reducing the occurrence of a series of symptoms and complications.

### Limitations

This is a single center retrospective analysis with a small sample size. Additionally, a patient’s physical condition is influenced by multiple factors. Therefore, this study cannot completely exclude the interference of confounding factors. Large-sample, multi-center prospective cohort studies are needed to further develop and improve TCM nutrition technology in the treatment of overweight PCOS patients.

## CONCLUSION

Nutrition intervention is a simple, inexpensive, and safe treatment method that analyzes the nutritional issues of patients through dialectical analysis and applies specific nutrition techniques to treat overweight PCOS patients. Combined with conventional drug treatment, TCM nutrition treatment can significantly improve glucose and lipid metabolism, hormone levels, and TCM syndrome of overweight PCOS patients, increase the ovulation and pregnancy rates, and reduce adverse reactions.

### Authors’ contributions:

**JF** conceived and designed the study. **YC, ZH and XW** collected the data and performed the analysis. **JF** was involved in the writing of the manuscript and is responsible for the integrity of the study. All authors have read and approved the final manuscript.
